# Patterns of access to reproductive health services in Ghana and Nigeria: results of a cluster analysis

**DOI:** 10.1186/s12889-020-08724-3

**Published:** 2020-04-23

**Authors:** Oluwasegun Jko Ogundele, Milena Pavlova, Wim Groot

**Affiliations:** 1 Department of Health Services Research; CAPHRI, Maastricht University Medical Center; Faculty of Health, Medicine and Life Sciences; Maastricht University, PO Box 616, 6200MD, Maastricht, The Netherlands; 2 United Nations University-Maastricht Economic and Social Research Institute on Innovation and Technology, Maastricht, The Netherlands

**Keywords:** Reproductive services, Maternal care, Family planning, Access, Nigeria, Ghana

## Abstract

**Background:**

Inequalities in access to health care result in systematic health differences between social groups. Interventions to improve health do not always consider these inequalities. To examine access to reproductive health care services in Ghana and Nigeria, the patterns of use of family planning and maternal care by women in these countries are explored.

**Methods:**

We used population-level data from the Ghana and Nigeria Demographic Health Surveys of 2014 and 2013 respectively. We applied a two-step cluster analysis followed by multinomial logistic regression analysis.

**Results:**

The initial two-step cluster analyses related to family planning identified three clusters of women in Ghana and Nigeria: women with high, medium and poor access to family planning services. The subsequent two-step cluster analyses related to maternal care identified five distinct clusters: higher, high, medium, low and poor access to maternal health services in Ghana and Nigeria. Multinomial logistic regression showed that compared to women with secondary/higher education, women without education have higher odds of poor access to family planning services in Nigeria (OR = 2.54, 95% CI: 1.90–3.39) and in Ghana (OR = 1.257, 95% CI: 0.77–2.03). Compared to white-collar workers, women who are not working have increased odds of poor access to maternal health services in Nigeria (OR = 1.579, 95% CI: 1.081–2.307, *p* ≤ 0.01). This association is not observed for Ghana. Household wealth is strongly associated with access to family planning services and maternal health care services in Nigeria. Not having insurance in Ghana is associated with low access to family planning services, while this is not the case in Nigeria. In both countries, the absence of insurance is associated with poor access to maternal health services.

**Conclusions:**

These differences confirm the importance of a focused context-specific approach towards reproductive health services, particularly to reduce inequality in access resulting from socio-economic status. Interventions should be focused on the categorization of services and population groups into priority classes based on needs assessment. In this way, they can help expand coverage of quality services bottom up to improve access among these vulnerable groups.

## Background

Inequalities in access to health care can result in health differences between social groups. Interventions to create universal access to health care and to improve health outcomes, do not always consider these inequalities. Women are exposed to unequal access to health care services globally [1]. This is particularly the case for reproductive health care services [2], which include contraceptives, maternal health services, and services related to sexual health [3–5]. Targets have been set to improve these services. For example, the Sustainable Development Goal 3 aims to ensure universal access to reproductive health care services [6].

Factors that determine access to reproductive health services are related to both demand and supply and can be divided into social and economic factors [7]. Education, occupation, wealth and possession of insurance, among others, are significant predictors of inequality in access to reproductive health services in Sub-Saharan Africa [8–13]. However, previous studies have mostly focused on the determinants of service use in a country or region [11, 14, 15]. There is a need for cross-country comparisons to shed light on similarities and/or dissimilarities between groups of users of reproductive health services in Sub-Saharan African countries.

This study examines access to reproductive health care services among women of reproductive age in Ghana and Nigeria. We use data from the Demographic Health Surveys (DHS) of Ghana carried out in 2014 and that of Nigeria carried out in 2013. The two countries are selected for this study based on the similarities in trends, health outcomes as well as data availability. At the same time, their health care systems are different. For example, Ghana has an established national health insurance system, while there is no such well-established system in Nigeria [16]. Ghana and Nigeria introduced a minimal user fee in the early 1970s, which was later abandoned in both countries due to cash crunches [17]. Ghana offers free-of-charge maternal care. The health insurance scheme in the country is reported to cover 65% of the population, which reduces the out-of-pocket health expenditure (66% of total health spending) [18]. One study using a cluster analysis method has shown that there are differences in the adequacy of maternal care available in Ghana and that there are disparities in the socio-demographic characteristics that determine access [8]. Insurance in Nigeria covers 3.5% of the population with out-of-pocket health expenditure amounting to over 90% of total health spending [17, 18]. These differences are expected to result in differences in access to reproductive health services, which we investigate in this paper.

## Methods

The DHS are nationally representative cross-sectional surveys carried out in low- and middle-income countries periodically [19, 20]. The DHS adopts a multi-stage sampling design. Samples selected for enumeration, are ensured to be representative and comparative across countries. The DHS of Ghana and Nigeria included in this study, involved a two-stage sampling procedure: first selecting the location and then, selecting households per location at random [19, 20]. Within a household, respondents were selected by gender for the different questionnaire types. A respondent was included if he/she was a usual member of the household or had spent the night preceding the survey, in the household.

We only used data for women of reproductive age (15–49 years) in Ghana and Nigeria who had given birth during the last 5 years before the survey and were able to provide information on the use of reproductive health services. Our study included 4142 women from the DHS of Ghana and 7725 women from the DHS of Nigeria. The indicators of reproductive care that we used in the cluster analysis are categorized as family planning services and maternal health services and are shown in Appendix A of the supplementary file.

We first performed two-step cluster analyses, which provided insight into the patterns of reproductive health services use among women of reproductive age in both countries. Cluster analysis is a method to classify similarities or dissimilarities based on respondents’ data [21]. Four cluster analyses were carried out, namely one for family planning services and another one for maternal health services for each of the two countries. The clusters generated by the cluster analysis procedure are shown in Appendix A in the supplementary file. In particular, the two-step clustering procedure uses the Schwarz’s Bayesian Information Criterion (BIC) method to determine the number of clusters. Different clustering solutions are compared and the clustering solution with the lowest BIC is selected by the procedure. We inspected this clustering and accept it as adequate. The stability and reliability of the cluster analyses were confirmed by repeating the clustering procedure produced no less than 10 times. The repeated analyses resulted in the same cluster quality. The two-step cluster analysis procedure specifies the clustering quality based on the Silhouette Index (SI). The SI indicates how well each subject/object lies within its cluster, and thus, it validates the clustering outcomes. The SI ranges from − 1 to 1. SI greater or equal to 0.5 indicates good clustering quality.

We titled the clusters based on the quality and adequacy of medical care used by women in each cluster compared to what is usually provided in government-licensed medical facilities. Thus, in the poor access cluster, on average, women reported using less and lower quality care than the care usually provided at government-licensed facilities, and in the high access cluster, women reported using more and better care. Details about the cluster composition variables, patterns, and quality are presented in Appendix A in the supplementary file.

Multinomial logistic regression was used to identify factors associated with the cluster membership determined during the cluster analyses. A total of four regression analyses were conducted. The cluster membership generated in each cluster analysis was the dependent variable in the multinomial logistic regression analyses. The explanatory variables consisted of women’s background characteristics that were found to be associated with the use of family planning services and maternal health services in previous studies and were available in our dataset. Sample weights were applied for the multinomial logistic regression. Software package SPSS version 23 was used for all data analyses.

## Results

Descriptive statistics on the socio-economic characteristics of the two samples and primary results of the two-steps cluster analyses can be found in the appendices (supplementary files). Below, we present the key findings of the cluster analyses as well as the results of the regression analyses.

### Cluster analysis

The two-step cluster analysis of family planning service use in Ghana automatically produced 3 distinct clusters. In the two-step cluster analysis of family planning service use in Nigeria, the number of clusters (3 clusters) was fixed in advance to be able to produce meaningful clusters. The clusters are presented in Table 1. The clusters were inspected and labeled as high, medium, and poor access to family planning services based on the services used by women in each cluster (see Methods section). The cluster with high access to family planning services captures 19.1 and 21.4% of women in Nigeria’s and Ghana’s sample respectively. The other extreme is the third cluster that consists of women whose access can be described as poor; 71.5% of women in Nigeria’s sample belong to this cluster and 64.2% of women in Ghana’s sample.

**Table 1:** Frequency distribution of cluster membership

	Family planning services			
	Ghana		Nigeria	
Cluster group	Obs	%	Obs	%
Poor-access	2755	64.2	5638	71.5
Medium Access	918	14.4	1507	9.3
High-Access	619	21.4	736	19.1
**Maternal health service**				
	Ghana		Nigeria	
	Obs	%	Obs	%
Low-access	293	7.1	346	4.4
Poor -Access	1053	25.4	1452	18.5
Medium-Access	756	18.2	2027	25.9
High-Access	952	23.0	1693	21.6
Higher-access	1092	26.3	2315	29.6

Note: The total percentage of Family planning in Nigeria does not add up to 100% due to approximation

We did not predefine the number of clusters for maternal health services. For both countries, the two-step cluster analyses of maternal health services use resulted in five clusters, which we inspected and labeled as higher, high, medium, low and poor access to maternal health services (see Table 1). The higher-access cluster captures 29.6% of women in Nigeria’s sample and 26.3% of the women in Ghana’s sample. Relative to the other four clusters, a larger proportion of members of this cluster report that they accessed government hospitals for antenatal care and used institutional maternal care more. The high-access cluster consists of 21.6% of women in Nigeria’s sample and 23.0% of women in Ghana’s sample. For both countries, this cluster has a lower proportion of women who accessed government health centers for antenatal care or got assistance from physicians during childbirth. Members of the medium-access cluster in both countries used private facilities for antenatal care as well as for childbirth. This cluster of women makes up 25.9% of Nigeria’s sample and 18.2% of the Ghana sample. Members of the low-access cluster in both countries mostly report that they accessed government health posts/dispensaries for antenatal care but did not have skilled assistance during childbirth. In Nigeria’s sample, 4.4% of women fall into this cluster and in the Ghana’s sample, this share is 7.1%. Lastly, 18.5 and 25.4% of women from the Nigeria’s and Ghana’s sample respectively are members of the poor-access cluster. Members of this cluster mostly did not receive institutionalized maternal care. For both countries, the poor-access cluster has a high proportion of members who had home childbirth and used traditional birth attendants during childbirth.

### Regression analysis

The dependent variables in the four multinomial logistic regressions were the four cluster membership variables generated in the cluster analyses. Tables 2 and 3 present the odds ratios for the four regressions. Information about the independent variables used and the full results of the regression analyses can be found in Appendix B of the supplementary file.

**Table 2:** Odds ratio of use of family planning services in Nigeria and Ghana (multinomial logistic regression)

Background characteristics	Nigeria		Ghana	
	Medium-access	Poor-access	Medium-access	Poor-access
	Reference category: High access		Reference category: High access	
	Exp B (95% CI)	Exp B (95% CI)	Exp B (95% CI)	Exp B (95% CI)
**Maternal age**	1.018^a^ (0.998–1.038)	1.015^b^ (1.001–1.029)	1.047^c^ (1.023–1.072)	1.037^c^ (1.019–1.055)
**Number of children alive**	0.940^a^ (0.878–1.007)	0.774^c^ (0.738–0.813)	0.851^c^ (0.777–0.931)	0.849^c^ (0.795–0.908)
**Marital status**				
Married (ref)	1	1	1	1
Never married	0.338^c^ (0.185–0.619)	0.550^c^ (0.395–0.767)	1.068 (0.705–1.617)	1.359^b^ (1.008–1.833)
Widowed/separated/divorced	1.038 (0.606–1.777)	1.531^b^ (1.052–2.228)	1.004 (0.656–1.535)	0.935 (0.695–1.258)
**Maternal Education**				
Secondary/ Higher (ref)	1	1	1	1
No education	1.257 (0.776–2.037)	2.544^c^ (1.907–3.395)	1.350 (0.941–1.938)	1.527^c^ (1.173–1.988)
Primary	0.825 (0.642–1.061)	1.111 (0.939–1.314)	0.817 (0.601–1.112)	0.961 (0.774–1.192)
**Maternal Occupation**				
White collar (ref)	1	1	1	1
Not working	1.006 (0.706–1.435)	1.135 (0.882–1.459)	1.732^b^ (1.027–2.921)	2.194^c^ (1.447–3.325)
Services and manual	1.134 (0.806–1.594)	1.283^b^ (1.002–1.642)	1.227 (0.723–2.081)	1.727^c^ (1.137–2.622)
Sales	0.930 (0.687–1.258)	0.987 (0.793–1.228)	1.238 (0.771–1.987)	1.686^c^ (1.152–2.466)
Agriculture	0.805 (0.503–1.291)	1.194 (0.867–1.646)	1.378 (0.785–2.421)	1.801^c^ (1.164–2.786)
**Household Wealth**				
Richest (ref)	1	1	1	1
Poorest	0.762 (0.220–2.633)	3.417^c^ (1.825–6.396)	0.947 (0.519–1.731)	1.403 (0.895–2.200)
Poorer	1.026 (0.621–1.694)	2.282^c^ (1.669–3.120)	0.781 (0.469–1.302)	1.148 (0.786–1.677)
Middle	1.241 (0.899–1.714)	1.979^c^ (1.583–2.475)	0.775 (0.508–1.183)	0.997 (0.723–1.375)
Richer	1.232^a^ (0.977–1.553)	1.704^c^ (1.448–2.006)	0.928 (0.652–1.32)	0.968 (0.734–1.277)
**Residence**				
Urban (ref)	1	1	1	1
Rural	1.092 (0.864–1.381)	0.927 (0.785–1.093)	0.748^a^ (0.556–1.006)	0.779^b^ (0.624–0.974)
**Has health insurance**				
Yes (ref)	1	1	1	1
No	0.909 (0.602–1.373)	1.374^b^ (1.011–1.867)	0.320^c^ (0.246–0.417)	0.829^b^ (0.699–0.983)
**Religion**				
Other Christian (ref)	1	1	1	1
Catholic	1.078 (0.811–1.433)	1.048 (0.849–1.295)	0.824 (0.57–1.19)	0.891 (0.676–1.173)
Traditionalist/ none	1.324 (0.254–6.898)	2.043 (0.625–6.678)	1.128 (0.75–1.695)	1.137 (0.840–1.538)
Islam	1.188 (0.915–1.542)	1.474^c^ (1.240–1.753)	0.527^b^ (0.297–0.932)	1.109 (0.781–1.575)
**Need permission for medical help**				
Not a big problem (ref)	1	1	1	1
Big problem	1.247 (0.771–2.016)	1.130 (0.792–1.611)	1.036 (0.64–1.678)	1.015 (0.705–1.462)
**Need money for medical help**				
Not a big problem (ref)	1	1	1	1
Big problem	1.041 (0.835–1.297)	0.812^c^ (0.697–0.947)	1.297^a^ (0.999–1.682)	1.051 (0.868–1.273)
**Distance to health facility**				
Not a big problem (ref)	1	1	1	1
Big problem	0.771^a^ (0.571–1.042)	1.109 (0.904–1.360)	1.237 (0.908–1.685)	1.164 (0.923–1.466)
**Do not want to visit health facility alone**				
Not a big problem (ref)	1	1	1	1
Big problem	1.728^b^ (1.112–2.685)	1.407^b^ (1.012–1.958)	1.017 (0.705–1.465)	1.020 (0.771–1.349)
**Heard family planning on radio last few months**				
Yes (ref)	1	1	1	1
No	0.885 (0.648–1.208)	0.800^b^ (0.643–0.997)	1.099 (0.848–1.424)	1.023 (0.846–1.236)
**Heard family planning on TV last few months**				
Yes (ref)	1	1	1	1
No	0.790^a^ (0.624–1.001)	1.090 (0.926–1.283)	0.827 (0.623–1.098)	1.012 (0.819–1.249)
**Heard family planning in print last few months**				
Yes (ref)	1	1	1	1
No	1.354^b^ (1.066–1.720)	1.105 (0.934–1.308)	0.552^b^ (0.327–0.933)	0.710 (0.458–1.103)
**Region (Nigeria)**				
South West (ref)	1	1	–	–
North Central	0.462^c^ (0.320–0.666)	0.962 (0.765–1.209)	–	–
North East	0.162^c^ (0.070–0.376)	1.574^b^ (1.101–2.250)	–	–
North West	0.038^c^ (0.016–0.086)	0.425^c^ (0.321–0.563)	–	–
South East	1.523^b^ (1.022–2.270)	1.390^b^ (1.01–0 1.913)	–	–
South South	1.100 (0.780–1.550)	0.965 (0.755–1.233)	–	–
**Region**				
Greater Accra (ref)	–	–	1	1
Western	–	–	1.742^b^ (1.097–2.765)	0.868 (0.610–1.234)
Central	–	–	0.754 (0.463–1.229)	0.691^b^ (0.494–0.966)
Volta	–	–	3.191^c^ (1.890–5.389)	0.499^c^ (0.326–0.764)
Eastern	–	–	0.474^c^ (0.273–0.825)	0.868 (0.617–1.223)
Ashanti	–	–	1.912^c^ (1.264–2.893)	1.103 (0.808–1.505)
Brong Ahafo	–	–	1.826^b^ (1.123–2.969)	0.658^b^ (0.451–0.960)
Northern	–	–	1.623 (0.835–3.155)	1.805^b^ (1.083–3.006)
Upper East	–	–	0.146^c^ (0.045–0.473)	0.709 (0.424–1.185)
Upper West	–	–	0.262^b^ (0.090–0.769)	0.728 (0.398–1.332)
**Ethnicity (Nigeria)**				
Yoruba (ref)	1	1	–	–
Other minorities	1.338^a^ (0.961–1.864)	1.953^c^ (1.566–2.436)	–	–
Fulani	1.961 (0.436–8.812)	3.352^c^ (1.699–6.612)	–	–
Igbo	2.134^c^ (1.471–3.096)	1.922^c^ (1.448–2.551)	–	–
Hausa	4.820^c^ (2.139–10.861)	11.842^c^ (7.766–18.059)	–	–
**Ethnicity (Ghana)**				
Akan (ref)	–	–	1	1
Ga/Dangme	–	–	1.294 (0.775–2.159)	1.142 (0.790–1.650)
Ewe	–	–	1.044 (0.687–1.584)	1.059 (0.780–1.437)
Guan	–	–	1.631 (0.783–3.396)	0.883 (0.469–1.664)
Mole-Dagbani	–	–	1.184 (0.754–1.860)	1.061 (0.754–1.493)
Grusi	–	–	0.942 (0.490–1.811)	0.682 (0.422–1.103)
Gurma	–	–	0.948 (0.523–1.720)	0.883 (0.563–1.385)
Mande	–	–	0.561 (0.266–1.181)	0.741 (0.446–1.230)
**Attitude of the health workers**				
Not a big problem (ref)	1	1	–	–
Big problem	0.900 (0.684–1.184)	1.147 (0.940–1.401)	–	–

^c^ p ≤ 0.01; ^b^ p ≤ 0.05; ^a^ p ≤ 0.10 (two-tailed test of significance)

**Table 3:** Odds ratio of use of maternal health service in Nigeria and Ghana: Ref: Higher access (multinomial logistic regression)

Background characteristics	Nigeria				Ghana			
	High-access	Medium-access	Low-access	Poor-access	High-access	Medium-access	Low-access	Poor-access
	Reference category: Higher access				Reference category: Higher access			
	Exp B (95% CI)	Exp B (95% CI)	Exp B (95% CI)	Exp B (95% CI)	Exp B (95% CI)	Exp B (95% CI)	Exp B (95% CI)	Exp B (95% CI)
**Maternal age**	0.977^c^ (0.963–0.992)	0.980^c^ (0.967–0.994)	0.956^c^ (0.930–0.982)	0.965^c^ (0.950–0.980)	0.977^b^ (0.957–0.997)	0.984^a^ (0.963–1.005)	1.020 (0.995–1.047)	0.961^c^ (0.940–0.983)
**Number of children alive**	1.101^c^ (1.045–1.16)	1.087^c^ (1.033–1.145)	1.113^b^ (1.016–1.220)	1.126^c^ (1.067–1.188)	1.105^b^ (1.016–1.201)	1.088^a^ (0.998–1.185)	1.000 (0.899–1.113)	1.239^c^ (1.134–1.353)
**Marital status**								
Married (ref)	1	1	1	1	1	1	1	1
Never married	1.081 (0.722–1.619)	0.894 (0.600–1.332)	1.550 (0.676–3.551)	1.382 (0.922–2.072)	0.981 (0.699–1.377)	0.713^a^ (0.495–1.026)	0.970 (0.628–1.498)	0.880 (0.604–1.281)
Widowed/separated/divorced	1.252 (0.871–1.800)	1.055 (0.729–1.527)	1.248 (0.634–2.459)	1.084 (0.740–1.588)	0.978 (0.676–1.413)	0.869 (0.59–1.278)	0.803 (0.477–1.350)	0.880 (0.591–1.310)
**Maternal Education**								
Secondary/ Higher (ref)	1	1	1	1	1	1	1	1
No education	1.145 (0.902–1.454)	0.690^c^ (0.524–0.909)	1.849^c^ (1.247–2.742)	1.431^c^ (1.132–1.809)	0.961 (0.702–1.316)	0.962 (0.691–1.341)	0.680^a^ (0.43–1.076)	1.542^c^ (1.115–2.132)
Primary	1.034 (0.855–1.251)	0.921 (0.763–1.111)	1.786^c^ (1.247–2.557)	1.387^c^ (1.140–1.687)	0.975 (0.742–1.280)	1.202 (0.914–1.580)	0.594^b^ (0.398–0.886)	1.380^b^ (1.036–1.838)
**Maternal Occupation**								
White collar (ref)	1	1	1	1	1	1	1	1
Not working	1.052 (0.779–1.421)	1.097 (0.847–1.422)	1.754 (0.752–4.093)	1.579^b^ (1.081–2.307)	1.074 (0.644–1.79)	0.771 (0.474–1.254)	0.889 (0.535–1.476)	1.076 (0.500–2.313)
Services and manual	1.492^c^ (1.109–2.006)	1.215 (0.940–1.570)	2.422^b^ (1.032–5.686)	1.719^c^ (1.172–2.522)	1.168 (0.699–1.952)	0.782 (0.479–1.276)	0.437^c^ (0.252–0.757)	1.141 (0.527–2.468)
Sales	1.289^a^ (0.979–1.698)	1.300^b^ (1.032–1.638)	2.729^b^ (1.198–6.219)	2.001^c^ (1.395–2.871)	0.824 (0.511–1.329)	0.576^b^ (0.368–0.903)	0.628^b^ (0.4–0.987)	0.779 (0.371–1.636)
Agriculture	1.697^c^ (1.187–2.428)	1.298 (0.922–1.826)	4.253^c^ (1.741–10.392)	1.667^b^ (1.076–2.584)	1.781^b^ (1.022–3.104)	0.735 (0.426–1.266)	0.539^a^ (0.277–1.05)	1.226 (0.561–2.682)
**Household Wealth**								
Richest (ref)	1	1	1	1	1	1	1	1
Poorest	4.726^c^ (2.982–7.489)	1.531 (0.855–2.740)	3.230^c^ (1.668–6.255)	6.592^c^ (4.247–10.233)	3.732^c^ (2.171–6.415)	3.826^c^ (2.16–6.775)	1.889 (0.863–4.135)	20.631^c^ (10.086–42.199)
Poorer	2.750^c^ (2.018–3.748)	1.104 (0.796–1.533)	1.580^a^ (0.921–2.711)	2.408^c^ (1.75–3.312)	2.514^c^ (1.605–3.938)	2.351^c^ (1.479–3.737)	1.679^a^ (0.949–2.97)	10.228^c^ (5.352–19.544)
Middle	1.829^c^ (1.433–2.334)	0.832 (0.657–1.054)	1.268 (0.795–2.023)	1.810^c^ (1.401–2.338)	1.967^c^ (1.363–2.838)	1.684^c^ (1.153–2.459)	1.077 (0.705–1.645)	6.376^c^ (3.508–11.587)
Richer	1.476^c^ (1.221–1.785)	0.733c (0.616–0.873)	0.665^a^ (0.433–1.022)	1.237^b^ (1.006–1.522)	1.263 (0.926–1.723)	1.354^a^ (0.991–1.851)	0.627^c^ (0.447–0.881)	1.328 (0.713–2.474)
**Residence**								
Urban (ref)	1	1	1	1	1	1	1	1
Rural	0.654^c^ (0.550–0.779)	1.086 (0.914–1.290)	0.539^c^ (0.384–0.757)	0.817^b^ (0.678–0.985)	1.149 (0.889–1.483)	1.303^a^ (0.999–1.699)	0.646^b^ (0.453–0.921)	2.139^c^ (1.600–2.86)
**Has health insurance**								
Yes (ref)	1	1	1	1	1	1	1	1
No	1.167 (0.770–1.770)	0.789 (0.574–1.085)	0.563 (0.274–1.156)	1.570^a^ (0.926–2.661)	1.146 (0.923–1.422)	1.260^b^ (1.009–1.573)	1.209 (0.919–1.590)	1.888^c^ (1.500–2.376)
**Religion**								
Other Christian (ref)	1	1	1	1	1	1	1	1
Catholic	1.244^a^ (0.970–1.595)	1.582^a^ (1.269–1.973)	1.355 (0.847–2.166)	0.952 (0.709–1.278)	0.710^b^ (0.505–0.998)	0.698^b^ (0.492–0.99)	0.496^c^ (0.300–0.820)	0.679^b^ (0.470–0.980)
vTraditionalist/ none	0.643 (0.249–1.661)	0.470 (0.165–1.340)	0.379 (0.034–4.263)	0.470 (0.154–1.432)	1.031 (0.719–1.478)	0.763 (0.524–1.113)	0.825 (0.500–1.363)	0.781 (0.528–1.154)
Islam	0.992 (0.810–1.214)	0.916 (0.760–1.104)	1.429^a^ (0.971–2.105)	0.976 (0.783–1.215)	1.112 (0.644–1.921)	1.294 (0.724–2.314)	2.472^c^ (1.263–4.837)	2.244^c^ (1.351–3.726)
**Need permission for medical help**								
Not a big problem (ref)	1	1	1	1	1	1	1	1
Big problem	0.990 (0.709–1.384)	0.781 (0.540–1.128)	0.620 (0.312–1.232)	1.016 (0.730–1.415)	1.097 (0.711–1.691)	0.816 (0.525–1.267)	0.478^b^ (0.23–0.993)	0.840 (0.532–1.328)
**Need money for medical help**								
Not a big problem (ref)	1	1	1	1	1	1	1	1
Big problem	0.996 (0.843–1.178)	0.879 (0.744–1.037)	1.033 (0.768–1.389)	1.304^c^ (1.100–1.547)	0.999 (0.794–1.256)	1.239^a^ (0.977–1.572)	0.668^b^ (0.487–0.916)	0.889 (0.693–1.14)
**Distance to health facility**								
Not a big problem (ref)	1	1	1	1	1	1	1	1
Big problem	1.211^a^ (0.974–1.506)	1.652^c^ (1.327–2.057)	1.109 (0.760–1.620)	1.510^c^ (1.216–1.876)	0.756^a^ (0.569–1.005)	0.971 (0.729–1.292)	1.315 (0.899–1.924)	1.099 (0.827–1.461)
**Do not want to visit health facility alone**								
Not a big problem (ref)	1	1	1	1	1	1	1	1
Big problem	0.869 (0.632–1.196)	0.982 (0.714–1.351)	0.859 (0.471–1.565)	0.791 (0.566–1.106)	1.020 (0.728–1.427)	0.932 (0.658–1.321)	0.930 (0.592–1.462)	1.431^b^ (1.02–2.008)
**Heard family planning on radio last few months**								
Yes (ref)	1	1	1	1	1	1	1	1
No	1.008 (0.797–1.275)	0.776^b^ (0.612–0.983)	0.418^c^ (0.240–0.730)	0.546^c^ (0.419–0.712)	1.180 (0.937–1.487)	1.515^c^ (1.193–1.925)	1.314^a^ (0.968–1.784)	1.212 (0.946–1.554)
**Heard family planning on TV last few months**								
Yes (ref)	1	1	1	1	1	1	1	1
No	0.917 (0.768–1.095)	0.934 (0.785–1.112)	0.928 (0.670–1.286)	1.075 (0.896–1.290)	1.090 (0.848–1.399)	0.852 (0.656–1.105)	0.773 (0.554–1.077)	0.923 (0.704–1.21)
**Heard family planning in print last few months**								
Yes (ref)	1	1	1	1	1	1	1	1
No	0.861 (0.708–1.047)	0.877 (0.729–1.053)	1.384 (0.885–2.165)	1.068 (0.861–1.324)	1.733^b^ (1.042–2.883)	0.930 (0.588–1.473)	1.526 (0.909–2.562)	1.195 (0.614–2.324)
**Region (Nigeria)**								
South West (ref)	1	1	1	1	–	–	–	–
North Central	0.377^c^ (0.286–0.499)	0.335^c^ (0.264–0.425)	0.666 (0.381–1.166)	0.343^c^ (0.250–0.470)	–	–	–	–
North East	0.427^c^ (0.302–0.603)	0.061^c^ (0.039–0.096)	1.162 (0.632–2.136)	0.596^c^ (0.418–0.850)	–	–	–	–
North West	0.206^c^ (0.145–0.292)	0.030^c^ (0.020–0.046)	0.344^c^ (0.178–0.665)	0.346^c^ (0.242–0.492)	–	–	–	–
South East	1.112 (0.736–1.681)	0.771 (0.554–1.073)	1.712 (0.635–4.618)	0.961 (0.567–1.629)	–	–	–	–
South South	1.031 (0.767–1.387)	0.319^c^ (0.244–0.417)	0.248^a^ (0.107–0.577)	1.401^b^ (1.019–1.928)	–	–	–	–
**Region**								
Greater Accra (ref)	–	–	–	–	1	1	1	1
Western	–	–	–	–	0.369^c^ (0.237–0.576)	1.444^a^ (0.948–2.198)	1.013 (0.625–1.64)	1.340 (0.767–2.342)
Central	–	–	–	–	0.994 (0.666–1.483)	1.596^b^ (1.036–2.459)	1.171 (0.721–1.904)	2.101^c^ (1.211–3.648)
Volta	–	–	–	–	1.133 (0.695–1.849)	0.480^c^ (0.274–0.841)	0.411^c^ (0.208–0.811)	0.834 (0.452–1.539)
**Region (continued)**								
Eastern	–	–	–	–	0.444^c^ (0.288–0.684)	1.228 (0.804–1.874)	0.570^b^ (0.333–0.977)	1.253 (0.733–2.142)
Ashanti	–	–	–	–	0.900 (0.633–1.279)	2.429^c^ (1.681–3.511)	1.660^c^ (1.131–2.435)	1.093 (0.633–1.888)
Brong Ahafo	–	–	–	–	0.648^a^ (0.415–1.01)	1.705^b^ (1.075–2.705)	1.690^b^ (1.014–2.818)	0.871 (0.478–1.587)
Northern	–	–	–	–	2.036^b^ (1.163–3.564)	0.753 (0.382–1.484)	0.225^b^ (0.071–0.711)	2.962^c^ (1.489–5.891)
Upper East	–	–	–	–	3.661^c^ (1.92–6.98)	1.161 (0.542–2.486)	0.762 (0.249–2.329)	0.665 (0.287–1.541)
Upper West	–	–	–	–	1.789 (0.713–4.489)	7.466c (3.195–17.443)	0.348 (0.033–3.654)	2.861^b^ (1.079–7.586)
**Ethnicity (Nigeria)**								
Yoruba (ref)	1	1	1		–			
Other minorities	0.541^c^ (0.412–0.71)	0.987 (0.780–1.249)	1.223 (0.677–2.208)	0.816 (0.603–1.105)	–	–	–	–
Fulani	0.681 (0.405–1.143)	0.868 (0.400–1.883)	0.897 (0.376–2.139)	0.881 (0.529–1.466)	–	–	–	–
Igbo	0.759 (0.519–1.109)	1.808^c^ (1.336–2.447)	1.014 (0.378–2.722)	0.495^c^ (0.305–0.803)	–	–	–	–
Hausa	0.862 (0.59–1.259)	1.177 (0.760–1.822)	2.231^b^ (1.122–4.438)	1.416^a^ (0.965–2.078)	–	–	–	–
**Ethnicity (Ghana)**								
Akan (ref)	–	–	–	–	1	1	1	1
Ga/Dangme	–	–	–	–	1.592^b^ (1.068–2.374)	1.286 (0.813–2.035)	1.456 (0.892–2.376)	1.319 (0.776–2.243)
Ewe	–	–	–	–	0.647^b^ (0.438–0.957)	1.469^b^ (1.019–2.119)	1.633^b^ (1.076–2.477)	1.154 (0.764–1.743)
Guan	–	–	–	–	1.544 (0.676–3.525)	3.760^c^ (1.678–8.424)	7.107^c^ (3.037–16.632)	1.639 (0.674–3.988)
Mole-Dagbani	–	–	–	–	0.754 (0.494–1.150)	1.391^a^ (0.927–2.088)	1.605^a^ (0.955–2.698)	1.056 (0.676–1.649)
Grusi	–	–	–	–	0.850 (0.463–1.561)	1.102 (0.587–2.07)	1.056 (0.441–2.527)	1.113 (0.564–2.199)
Gurma	–	–	–	–	0.795 (0.438–1.443)	1.249 (0.658–2.373)	0.929 (0.33–2.615)	1.438 (0.792–2.613)
Mande	–	–	–	–	0.604 (0.318–1.149)	1.257 (0.651–2.425)	0.840 (0.34–2.071)	1.252 (0.627–2.497)
**Attitude of the health workers**								
Not a big problem (ref)	1	1	1	1	–	–	–	–
Big problem	1.707^c^ (1.314–2.217)	1.361^c^ (1.098–1.686)	0.809 (0.450–1.455)	2.495^c^ (1.748–3.56)	–	–	–	–

^c^*p* ≤ 0.01; ^b^*p* ≤ 0.05; ^a^*p* ≤ 0.10 (two-tailed test of significance

For family planning services, the results in Table 2 show that in both countries, women with no education, compared to women with secondary or higher education, have higher odds to belong to the poor-access family planning cluster (in Nigeria OR = 2.544, 95% CI:1.907–3.395, *p* ≤ 0.01 and in Ghana OR = 1.527, 95% CI: 1.173–1.988, *p* ≤ 0.01). Increased odds of having poor-access to family planning services are found for women in Ghana who do not belong to white-collar workers but not among women who live in rural areas, and also not among women in any of the wealth quintiles. Higher odds of poor-access to family planning services are also found for women in Nigeria who belong to the service-occupational category (OR = 1.283, 95% CI: 1.002–1.642, *p* ≤ 0.05), compared with white-collar workers. The odds of poor-access are as much as three times higher among the poorest quintile (95% CI: 1.825–6.396, *p* ≤ 0.01) than the richest quintile; and among those who have no insurance (OR = 1.374, 95% CI: 1.011–1.867, *p* ≤ 0.05) compared to those with insurance.

Table 3 shows the regression results on access to maternal health services in Nigeria and Ghana. In Nigeria’s sample, women with primary or no education have higher odds to have poor-access (OR = 1.387, 95% CI: 1.140–1.687, *p* ≤ 0.01) or low-access (OR = 1.786, 95% CI: 1.247–2.557, *p* ≤ 0.01) to maternal health services. In Nigeria’s sample, women who are not working have higher odds to belong to the cluster of poor-access maternal health services only (OR = 1.579, 95% CI 1.081–2.307, *p* ≤ 0.01). Compared to women in the white-collar occupational group, women in other occupational categories in Nigeria also have higher odds to belong to the poor-access cluster. Women in other occupational categories in Nigeria also have higher odds to belong to the poor-access cluster. Women in all household wealth quintiles have higher odds to have high- or poor-access to maternal health services; women without insurance have higher odds to have high or poor-access to maternal health services. Results for Ghana show that women with primary (OR = 1.38, 95% CI: 1.036–1.838, *p* ≤ 0.05) or no education (OR = 1.542, 95% CI: 1.115–2.132, *p* ≤ 0.01) have higher odds of poor-access to maternal health services. Only women in the agriculture occupational group have higher odds of high-access to maternal health services compared to women in the white-collar sector. Women without health insurance have higher odds of access to maternal health care services.

## Discussion

As shown by our results, access to reproductive health services varies among women of reproductive age in Ghana and Nigeria. A large proportion of women in Ghana’s and Nigeria’s samples have poor access to family planning services. Most women do not have access to modern contraceptives. They use traditional birth control methods and do not have the means for needed services. These differences in access to maternal health services in both countries reflect a broader gap in health care use between women who access antenatal care at government hospitals for childbirth with a physician present and women who are not able to access such services. In particular, women in the low-access cluster are restricted to services at government health posts without skilled assistance during childbirth, or to services of antenatal care private vendors. This confirms that among women of reproductive age in Ghana and Nigeria, there is unequal access to reproductive health services.

This suggests a dysfunctional organization structure that creates constraints to use preventive and medical procedures provided by well-trained professionals [4, 18, 22].

Our results show that educational attainment is associated with access to family planning and maternal health services. Low educational attainment reduces the ability to overcome access barriers, particularly to maternal health services. This finding supports similar results in other studies on the importance of education in improving access to reproductive health services [10–12, 23]. Our results indicate that some women with low education intend to use contraceptives later while others use traditional contraceptive methods of family planning. Notably, the cluster with poor access to family planning services for a large part consists of women who have no intention of future contraceptive use. Lower-educated women seem to be less able to act on their intentions due to difficulties in overcoming access barriers or limited knowledge about the benefits of family planning [24]. The connection between education and socio-economic status could also explain this observation because low education attainment usually implies less access to resources [10, 25, 26]. This result further confirms what is known about the educational level as an indirect predictor of access to health care services [9].

Our results suggest that wealth/finance related inequality in access to reproductive health services is prominent in both Nigeria and Ghana. Considering finance-related inequality between the two countries, we find that women without insurance coverage in Ghana are less likely to access family planning services. This is dissimilar when compared to women without insurance in Nigeria; women in Nigeria who have poor-access to family planning opt for services such as traditional methods of contraception. These findings are consistent with other studies on the use of family planning services in the two countries and other parts of Africa [10]. This can be partially attributed to the inaccessibility of family planning services due to a cost-reducing scheme, which inadvertently increases the preference for traditional contraceptives among some women [27]. Another study also found a situation similar to Ghana among women in Burkina Faso and concluded that affordability of insurance premium varies by household income [13]. The poor access to reproductive health services in any of the wealth quintiles in Nigeria is expected considering the lack of insurance. The low coverage of insurance schemes such as the NHIS, particularly among informal workers or uneducated women, magnifies the effect of household wealth [17, 18].

There is an association between maternal occupation and access to maternal health services in both countries. Other studies have also reported associations between care use and occupation [14, 22, 23]. However, where associations between maternal occupation and access to reproductive health services are observable, disparity by type of livelihood is not unusual [22]. We observe such differences between Ghana and Nigeria as well. In particular, the group of white-collar workers seems to have better access to family planning services in Ghana but no such differences are found for maternal care. The results for Nigeria are just the opposite; occupation does not explain poor access to family planning services in Nigeria but white-collar workers seem to have access to maternal care. In Nigeria, the cost of maternal health services has to be endured by women themselves while in Ghana, these services are available to women through the free maternal care policy [28, 29]. Out-of-pocket payments for health have been consistently high in Nigeria compared to those in Ghana while insurance coverage is better in Ghana, particularly in the informal sector [27, 30].

This study has some limitations that need to be acknowledged. There was not much variation in some response variables and they had to be excluded from the analysis. The inclusion of country-specific variables helps to better reflect the women’s situation but this also creates some dissimilarities in the countries’ analytical models.

## Conclusion

This study provided evidence on inequalities in access to reproductive health services in Ghana and Nigeria. A key observation is the varied composition of services available for use at different access levels. Several imperative factors contribute to inequality in access to these services. After controlling for the effects of maternal-related variables, findings showed significant inequalities by educational attainment, household wealth, insurance status and woman’s occupational type. Much of the inequality in access to family planning services that are seen in Nigeria and Ghana is related to education. The contribution of household wealth and insurance status in creating unequal access was also evidenced in the study. Health programs, which seek to stimulate the use of reproductive health services in Ghana and Nigeria, could take into account the variation in access reported in this study to assure the user-centeredness of these programs. It is important to identify and prioritize services for the needs of vulnerable groups.

## Supplementary information

**Additional file 1.**

## Data Availability

The data used for this study can be accessed through the following link: https://dhsprogram.com/data/dataset_admin/login_main.cfm?CFID=15554769&CFTOKEN=6d6c1572de0c435a-5BA70A19-BD9F-EDBD-DBC0BB52339CAF30
