# Supplementary material for: Patterns of access to reproductive health services in Ghana and Nigeria: results of a cluster analysis
Source: BMC Public Health. 2020 Apr 23;20:549. doi: 10.1186/s12889-020-08724-3 (PMC7178999; doi:10.1186/s12889-020-08724-3)

**APPENDIX (supplementary material)**

**APPENDIX B**

*Structure of family planning Revealed Cluster: Nigeria*


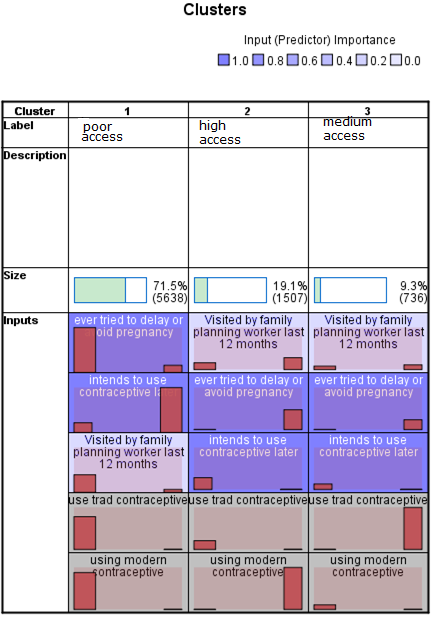


*Structure of family planning revealed cluster: Ghana*


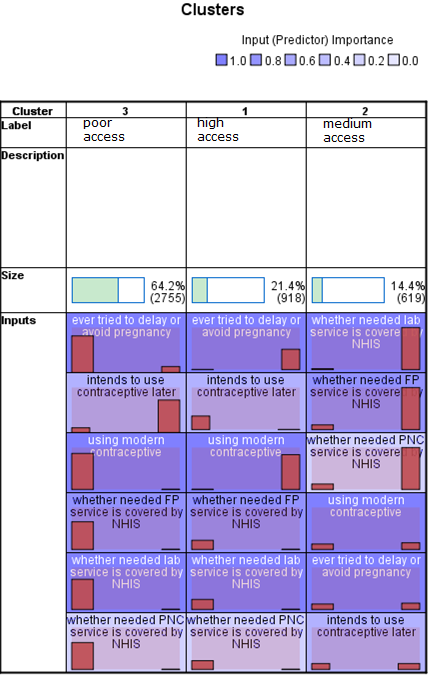


*Structure of maternal healthcare services revealed cluster: Nigeria*


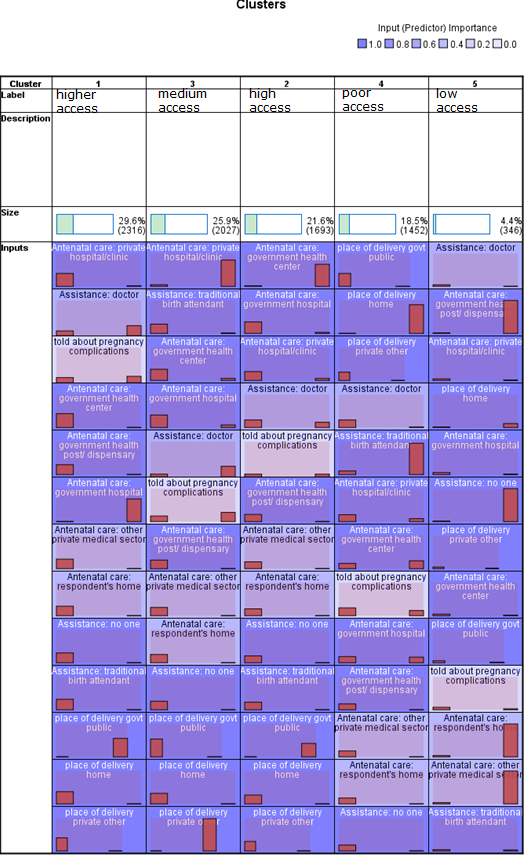


*Structure of maternal healthcare services revealed cluster: Ghana*


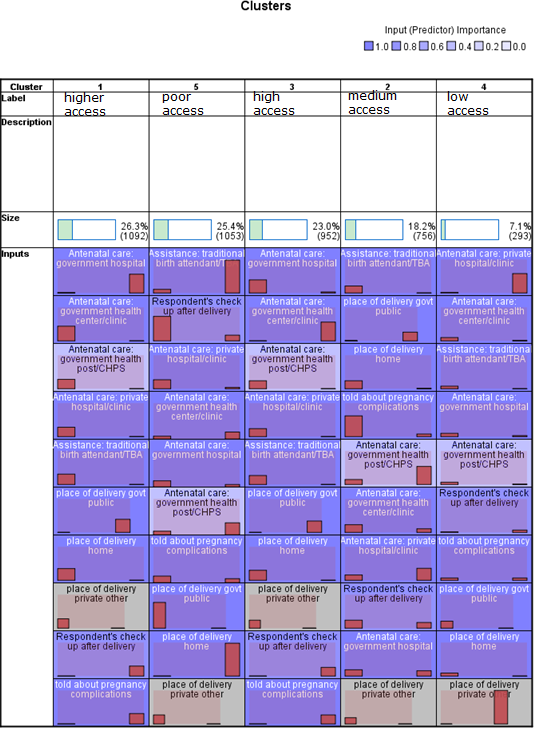

Supplement: Supplementary file 1 — Additional file 1. [file 12889_2020_8724_MOESM1_ESM.docx]
